# “We throw away an incredible amount of unused medicines“: Community pharmacy staff perspectives on environmental responsibility

**DOI:** 10.1016/j.rcsop.2025.100662

**Published:** 2025-09-19

**Authors:** Johanna Villén, Nicole Ljungdahl, Björn Wettermark, Helle Håkonsen, Marmar Nekoro, Sofia Kälvemark Sporrong

**Affiliations:** a Department of Pharmacy, Faculty of Pharmacy, Uppsala University, Uppsala, Sweden; b Pharmacy and Pharmacology Center, Institute of Biomedical Science, Faculty of Medicine, Vilnius University, Vilnius, Lithuania; c School of Public Health and Community Medicine, Institute of Medicine, University of Gothenburg, Gothenburg, Sweden; d Swedish Medical Products Agency, Sweden

**Keywords:** Environment, Pharmaceutical pollution, Community pharmacy, Pharmacists, Sweden

## Abstract

**Background:**

Pharmaceutical pollution is an increasing environmental concern. As key actors in the healthcare chain, community pharmacies play an important role in managing pharmaceuticals from an environmental perspective. Although environmental initiatives exist at the corporate level in Sweden, it remains unclear how well these are known to, or implemented by, pharmacy staff.

**Objectives:**

To explore how community pharmacy staff in Sweden perceive their role in reducing pharmaceutical pollution, and identify the opportunities and challenges they face in promoting environmentally friendly use of pharmaceuticals.

**Methods:**

Focus group discussions and individual semi-structured interviews were conducted with pharmacy staff in Sweden between May 2024 and February 2025. All focus group discussions and interviews were audio recorded, transcribed, and analysed using inductive qualitative content analysis.

**Results:**

Four focus group discussions and five individual interviews were conducted. The analysis resulted in three main categories. First, while there was an awareness of environmental issues, participants expressed a lack of concrete guidance and requested more information, such as lists of environmentally harmful pharmaceuticals. Second, pharmaceutical waste, originating both from households and pharmacies, was highlighted as a major concern. Third, there appeared to be a sense of limited agency and a wish for support from, for example, regulatory authorities to address pharmaceutical pollution.

**Conclusion:**

Swedish pharmacy staff view themselves as potential contributors to reducing pharmaceutical pollution, but struggle to identify concrete actions. They are concerned about pharmaceutical waste and responsibility is often shifted to other stakeholders.

## 1 Introduction

Pharmaceuticals play a crucial role in healthcare to prevent and cure diseases while improving quality of life. However, the widespread use of pharmaceuticals also brings significant environmental challenges, as residues in water and soil can disrupt ecosystems and pose a risk to human health.^1^^,^^2^ The release of pharmaceuticals into the environment has substantial negative consequences, including the development of antibiotic resistance and disruptions of entire ecosystems.^3^ A well-documented example of the ecological impact of pharmaceutical contamination occurred in the 1990s, when vulture populations in India and Pakistan experienced dramatic declines due to consumption of livestock carcasses treated with diclofenac.^4^ Other examples involve 17α-ethinyl estradiol and levonorgestrel, hormones used in oral contraceptives, which have been associated with adverse reproductive effects in aquatic species, including feminisation and population declines in fish, as well as impaired ovarian development and fertility in frogs following early-life exposure.5., 6., 7.

In Europe, pharmaceutical pollution primarily results from urban wastewater, resulting from the use and disposal of pharmaceuticals in humans.^2^ To address the environmental risks posed by pharmaceutical pollution, the concept of “green pharmacy” has emerged as a strategy.^8^^,^^9^ Green pharmacy refers to a set of practices aimed at reducing pharmaceutical pollution throughout the entire life cycle of pharmaceuticals, from research and development through manufacturing, prescribing, and dispensing to disposal.^8^ Interest in these practices has grown, with increasing calls for pharmacists to take a more active role in minimising their environmental impact as part of broader sustainability efforts.10., 11., 12. Notably, in 2016, the International Pharmaceutical Federation (FIP) introduced a roadmap for the development of green pharmacy, advocating for pharmacist-led initiatives to promote environmentally conscious pharmacy practices.^13^ In 2024, FIP published a Green Pharmacy Toolkit developed by the Royal Dutch Pharmacists Association, providing pharmacists with guidance on how to make pharmacies more sustainable.^14^

Several studies have highlighted the significance of pharmacist-led initiatives, such as patient education programs on the proper disposal of pharmaceuticals and strategies to minimise pharmaceutical waste.^12^^,^15., 16., 17., 18., 19. However, limited attention has been given to the perceptions and practices of community pharmacy staff in addressing pharmaceutical pollution. While guidelines often exist for safe disposal of unused pharmaceuticals, there are fewer guidelines or industry standards outlining how pharmacies should contribute to promoting more environmentally friendly use of pharmaceuticals on a broader scale.

Some research has explored the role of hospital pharmacists and their perspectives on environmental and climate issues.^12^^,^^20^^,^^21^ These studies have found that hospital pharmacists are generally positive toward considering environmental aspects in their work. However, they often lack the necessary knowledge and tools to effectively integrate these considerations into their practice. In contrast, community pharmacists, who interact directly with the public and play a key role in the use of pharmaceuticals at the individual level, have received less attention.^12^^,^^15^^,^^22^^,^^23^ Given their unique position at the intersection of healthcare provision and consumer behaviour, community pharmacists are well-placed to address pharmaceutical pollution, both through their direct involvement in dispensing pharmaceuticals and by raising awareness among patients and the public. The paradox that pharmaceuticals intended to promote health may also pose environmental risks that ultimately threaten human and ecological health is something that pharmacists must navigate and are in a unique position to influence.

This study aims to explore how community pharmacy staff perceive their role in reducing pharmaceutical pollution and identify the opportunities and challenges they face in promoting the environmentally conscious use of pharmaceuticals.

## 2 Method

A qualitative study was conducted using focus groups comprising non-managerial community pharmacy staff, and individual interviews with community pharmacy managers in Sweden. Focus groups were used as the main data collection method as they effectively generate rich data in areas that have not been extensively explored.^24^ Pharmacy managers were included in the study, but to avoid potential power imbalances in group discussions, they were interviewed individually. The Consolidated Criteria for Reporting Qualitative Research (COREQ)^25^ were used to guide the presentation of the study.

This study explores environmental responsibility in the context of community pharmacies. A deliberately open view of “environmental responsibility” is adopted, with a primary focus on the environmental impact of pharmaceutical substances. The interest is directed toward aspects most closely related to pharmacy practice around pharmaceuticals, their use, and what happens when they are no longer needed.

### 2.1 Setting

The study was conducted in Sweden, where there are around 1400 brick-and-mortar community pharmacies. The market is dominated by five major actors, of which one is online-only. Swedish pharmacies are required to ensure the availability of prescribed pharmaceuticals and provide advice on their safe and effective use. Additionally, they have self-selection sections for over-the-counter pharmaceuticals and related products. The pharmacy staff consists of approximately 50 % pharmacists and 22 % pharmacy technicians. Pharmacists in Sweden are educated at either bachelor's level or master's level. Both qualifications provide dispensing rights, allowing them to prepare and supply prescribed pharmaceuticals; however, neither has prescribing rights. Pharmacists and pharmacy technicians have complementary roles, as pharmacists are responsible for dispensing and ensuring the clinical appropriateness of prescribed pharmaceuticals, while pharmacy technicians primarily focus on giving self-care advice. Additional pharmacy staff include pharmacy assistants who manage tasks such as operating the register and handling inventory.^26^

Sweden's profit system for pharmacies is structured around a pharmacy margin model, which combines a fixed fee with a percentage of the pharmaceutical's price for prescription pharmaceuticals.^27^ Pharmacies receive additional profit for generic substitution.^26^ Sweden applies a system that mandates pharmacies to dispense the designated preferred brand of the month (i.e. the lowest-cost equivalent), when generic alternatives are available.^28^

According to Swedish law (SFS 2009:366), community pharmacies are permitted to return medicinal products that have expired or have too short a shelf life to be dispensed to customers, provided that the products are otherwise intact and that the pharmacy applies routines ensuring that items with the earliest expiry date are sold first.^29^

As a part of their responsibility for environmental and safe pharmaceutical handling, community pharmacies in Sweden are required to accept unused and leftover pharmaceuticals from the public for safe disposal.^30^ To further strengthen the role of pharmacies in promoting sustainability, the Swedish Pharmacy Association implemented the ‘Well Selected’ label in 2021. This label comprises a symbol that indicates which over-the-counter pharmaceuticals meet the pharmacies' criteria for transparent manufacturing.^30^

### 2.2 Interview guide

A semi-structured interview guide (Supplementary table 1) was developed based on previous literature on initiatives to promote environmentally conscious pharmacy practice.^10^^,^^12^^,^^13^^,^^17^ The interview guide included open-ended questions exploring views on the environmental impact of pharmaceuticals and current practices. Environmental responsibility was treated as a sensitising concept rather than a fixed construct, with interviews and analysis naturally gravitating toward practices around the use and disposal of pharmaceuticals. Perspectives on the environmental or climate impact of pharmaceutical packaging and transport were not included in the interview guide. Participants were asked about how pharmacies could reduce environmental harm, the role of pharmacies in relation to other stakeholders such as physicians, pharmaceutical industry, and regulatory authorities, and the challenges in implementing more environmentally friendly practices. The interview guide was pilot-tested in a focus group with colleagues of the authors, all of whom were pharmacists with experience of working in community pharmacies, resulting in minor adjustments to the probes and the order of the questions.

### 2.3 Participants

This study includes both community pharmacy managers and non-managerial staff. When referring to all participants collectively, the term pharmacy staff is used. Purposive sampling with maximum variation was used to recruit pharmacy staff from the counties surrounding Stockholm and Uppsala. Recruitment was conducted by the first author, who visited community pharmacies and provided oral and written information about the study. Efforts were made to obtain a heterogeneous sample, including variation in pharmacy characteristics (e.g., size, location, and socio-demographics in the surrounding area) as well as staff characteristics such as education level, gender, and years of professional experience. Pharmacy staff willing to participate provided their contact information and were subsequently invited to one of the focus groups or an individual interview. A gift card of 200 SEK (18€ or 20 USD) was given to each participant.

### 2.4 Data collection

Recruitment and data collection took place between May 2024 and February 2025, either online, in person within the premises of Uppsala University, or in person at a pharmacy. The first author moderated all the interviews, and an assistant moderator (second author) was present for all focus groups and interviews except the last interview. An initial transcription was generated using Whisper,^31^ an AI-based speech recognition tool enabled through Uppsala University at UPPMAX, and subsequently manually reviewed and refined for accuracy. The concept of information power^32^ guided the determination of sample size. After nine focus group discussions and interviews, the data collected were judged to be sufficiently rich based on the focused aim, the specificity and heterogeneity of the purposive sample, as well as the quality and depth of the dialogues. After each focus group discussion or interview, the first and second authors discussed their impressions, including key themes that appeared most important to the interviewees and any reflections that emerged during the process. These impressions were documented in a reflexive journal.^33^

### 2.5 Data analysis

An inductive content analysis inspired by Krueger^24^ was conducted using NVivo® (version 20.7.2.1560), following these steps: First, the first author thoroughly read all transcripts to gain a comprehensive understanding of the data. Initial patterns, regularities, and contradictions were identified and coded by the first author, leading to the development of a preliminary coding scheme and overarching categories. To enhance trustworthiness, four transcripts (two focus groups and two individual interviews) were independently read and analysed by the last author. A consensus meeting was then held between the first and last authors to compare and discuss the coding, resulting in a revised coding scheme with overarching categories. Following this, the first author proceeded to code all transcripts according to the revised coding scheme. Multiple consensus meetings were held between the first and last authors, during which the coding scheme was iteratively refined as new codes, subcategories, and main categories were defined. This process continued until intercoder agreement was achieved. To further ensure the reliability of the analysis, the interpretations of the categories were reviewed and discussed within the author group at multiple meetings. Additionally, the second author, who served as the assistant moderator, audited the final code to ensure agreement. Representative quotes were selected to illustrate the identified categories. Each quote is accompanied by an anonymised identifier, where ‘R' denotes a focus group respondent and ‘PM’ denotes a pharmacy manager followed by a number indicating the individual or group affiliation.

### 2.6 The research team

The authors brought varied preunderstandings to the research process, shaped by their professional backgrounds, experiences with community pharmacy, environmental issues, and qualitative research. The first and second authors are pharmacists and PhD candidates in pharmacoepidemiology and social pharmacy, respectively. Both have previous experience working in community pharmacies, and the first author has a long-standing engagement with environmental issues. The third author is a pharmacist working in pharmacoepidemiology, with prior experience in community pharmacy but no specific focus on environmental aspects. The fourth author is a pharmacist with a background in social pharmacy, with research experience related to community pharmacy practice. The fifth author is a system ecologist and PhD candidate in pharmacoepidemiology, without a background in pharmacy, and contributed an environmental science perspective. The last author has a background in social sciences and is highly experienced in qualitative research, professional ethics, and community pharmacy research. This author had no prior contact with the participants and was not involved in data collection. All authors had some experience with qualitative research, and the last author provided methodological guidance throughout the study. All authors except two (fifth and last author) are pharmacists, and all but one (third author) are women.

### 2.7 Ethical considerations

Participation in the study was voluntary, and all participants were asked to give their informed consent before starting the focus group discussion or interviews. The transcripts were anonymised and treated confidentially so that no unauthorised persons could access them. The Swedish Ethical Review Authority was consulted and responded that they had no ethical objections to the research project (ref no 2023–01949-01).

## 3 Results

In total, four focus groups were conducted, each with two to six participants (Supplementary table 2). Two groups met in person at the premises of Uppsala University, the other two were conducted online. Each focus group lasted approximately 80 min (from 50 to 102 min) and was conducted in the evening. Five individual interviews were conducted with pharmacy managers, either online or in a room at a pharmacy and lasted approximately 30 min (from 25 to 37 min). A total of 19 participants were included in the study (Table 1). The majority were women, most held a university degree in pharmacy. The participants had a median of 9 years of pharmacy work experience. All four major pharmacy chains, with brick-and-mortar pharmacies in Sweden, were represented among the participants.

**Table 1:** Demographics of the participants.

Total (*n* = 19)	Category	
Gender (n)	Women	15
	Men	4
Level of pharmacy education (n)	No university education within the pharmacy field	2
	Bachelor of Pharmacy	11
	Master of Pharmacy	6
Pharmacy working experience (years)	Median	9 years
	Range	1–45 years

The analysis resulted in three main categories: 1) environmental awareness exists, but concrete guidance is lacking, 2) pharmaceutical waste, both from households and pharmacies, is a major issue, and 3) there is limited personal agency and a need for support to address pharmaceutical pollution. For an overview of subcategories and codes, see Table 2.

**Table 2:** Main categories, subcategories, and codes that emerged from the content analysis.

Category	Subcategory	Code
Environmental awareness exists, but concrete guidance is lacking	Awareness and willingness	• Diclofenac as an eye-opener
		• Education on pharmaceuticals in the environment
	Concrete guidance	• A wish for concrete guidance on environmentally harmful pharmaceuticals
Pharmaceutical waste is a major issue, both from households and pharmacies	Pharmaceutical waste from households	• Over-prescribing leads to unnecessary waste
		• Systemic incentives encourage excessive dispensing
		• Current efforts to reduce waste include counselling and guidance
		• Availability of starter packs
		• Potential improvements: reuse and recycling, as well as dispensing smaller packages
	Pharmaceutical waste from the pharmacy	• Stock requirements result in waste
		• Inventory management as a waste-reduction strategy
		• Potential improvement: call for greater flexibility in dispensing
Limited personal agency and a need for support	Regulatory support	• Key environmental decisions must be made at a higher level
	Responsibility of pharmacy chains	• Sustainability as a priority for pharmacy chains
		• Financial barriers
	Role of the pharmaceutical industry	• Manufacturing
	Physicians' role	• What and how much pharmaceuticals are prescribed
	Customer engagement	• Customer engagement
		• Need for public awareness campaigns

### 3.1 Environmental awareness exists, but concrete guidance is lacking

Participants in this study recognised the environmental impact of pharmaceuticals as a significant issue and expressed a *willingness* to take a more active role in addressing it. Almost all participants reported having received some form of, but limited, education on how pharmaceuticals affect the environment, especially in connection with the introduction of the transparency labelling for over-the-counter medicines (Well-selected). However, they still felt that their knowledge and therefore their ability to act was limited. Most were aware of the environmental impact of diclofenac, and some also mentioned antibiotics and hormones as examples of environmentally harmful pharmaceuticals. However, many participants expressed a need for more *concrete guidance,* such as lists displaying the pharmaceuticals with the highest environmental risk.

*“I know about antibiotics, hormones, diclofenac. But I can't explain how other medicines affect it [the environment] /…/. We who work in pharmacies need to get more information about it.” (Pm2).*

*“I would have liked some kind of database, just to have a better overview myself. There's no ready-made list of practical things, like ‘this is better than that because…’.” (R2:1).*

Pharmaceutical waste is a major issue, both from households and pharmacies.

Participants in this study perceived pharmaceutical waste as a major concern, with implications for the environment and economic efficiency. Among all topics discussed, pharmaceutical waste stood out as particularly important, it was the most frequently and spontaneously raised issue by the participants. They expressed clear frustration over the large quantities of unused pharmaceuticals being disposed of.

*“We throw away an incredible amount of unused medicines. We dispose of so much due to expiration dates. There's so much that could be done. It almost makes you feel sick to your stomach every month.” (R3:3).*

All participants mentioned that they often talk to customers about returning unused pharmaceuticals to the pharmacy. However, the participants saw it as important to reduce the need for disposal by ensuring that excess pharmaceuticals do not accumulate in the first place. They highlighted some factors contributing to this problem, with *overprescribing* being a recurring concern. Physicians were perceived to prescribe larger quantities of pharmaceuticals than patients needed, often due to a lack of individualised prescribing practices or a tendency to rely on pharmaceuticals as a “quick fix”.

*“- It feels like all parties profit from selling more medicines, and unfortunately, that's just the way it is. Another issue is that some conditions could be treated in ways other than with medicine. But in healthcare, they often choose to prescribe medicine instead. Maybe because it's cheaper to buy tablets than to pursue another treatment. /…/. But medicine is just the easier option. I think it's really strange how easily medicines are prescribed. (R4:1).*

*- It's kind of a ‘quick fix’, everyone is under time pressure, and everything is supposed to be as streamlined as possible. (R4:2)”.*

The participants also identified Sweden's patient reimbursement scheme^28^ as a contributing factor to pharmaceutical waste. While this system improves access to pharmaceuticals, they felt it could unintentionally encourage patients to claim prescriptions for pharmaceuticals they do not use. Once a patient reaches the maximum co-payment threshold, they do not pay for their pharmaceuticals for the rest of the year. This removes financial barriers, which may lead some individuals to stockpile and increase the risk of waste.

*“We notice that people sometimes come in with large bags full of completely unused medicines. The free card system that we have in Sweden [the reimbursement scheme] is great for those who have multiple illnesses and take expensive medicines. But on the other hand, quite a lot is stockpiled.” (Pm2).*

Additionally, non-manager respondents perceived that they were under pressure from their employer to *dispense all prescriptions* for a patient, further exacerbating the problem.

*“- It's part of our job to say, ‘You also have this and this prescription. Is there anything else you'd like to pick up?’ I mean, we are trained to ask and encourage every customer because they [pharmacy companies] measure everything /.../. We get weekly reminders, like, ‘Now the numbers have dropped, so you need to step up. You need to ask every customer if they have any other medicines to collect.’” (R4:1).*

To address these challenges with large amounts of pharmaceuticals being disposed of, participants emphasised the importance of *counselling and guidance* to customers as a strategy to minimise waste. This included giving advice on proper use of pharmaceuticals, discussing potential side effects, and questioning whether the customer needs to collect their entire prescription at once or at all.

*“When a customer comes in and says* ‘*I want everything*’*. I go through the list and ask,* ‘*Do you use all of this? You haven't picked this up since last summer. Is this something you actually use?*’ *Because that way, you reduce waste at the source, so to speak. That's where it's most important to intervene.” (R1:1).*

According to the participants, one contributing factor to the accumulation of leftover pharmaceuticals in households is that many pharmaceuticals are only available in larger *package sizes*, despite the fact that initial treatment phases or dosage adjustments often require smaller quantities. Respondents expressed a willingness to be able to break packages and dispense appropriate amounts (which is not allowed in Sweden). They noted that such measures could help patients avoid receiving unnecessary quantities of pharmaceuticals and, thereby, waste.

*“So, the idea is that pharmacies, or rather, pharmacists, should have the right to split a package of, say, 40 penicillin tablets and give 20 to a customer who only needs 20. And then keep the remaining tablets to give to another customer who also needs 20. /.../ Today, we have to open the package, take out the required amount, discard the rest, and only give the customer their prescribed 20 tablets…” (R1:1).*

One commonly mentioned barrier to reduce pharmaceutical waste was the limited availability of starter packs, which are small initial quantities of pharmaceuticals, intended to assess their suitability before larger packages are dispensed. Participants noted that these smaller packages are often more expensive than larger ones and not always readily available.

*“- One would like to push down the prices of starter packs. Because they are more expensive than large packages. The customer doesn't want to have a small pack that is more expensive than a large one. (R3:3).*

*- I agree. It would have been better if you could dispense less. Just when they are about to start something. Too often they may not be able to handle it. And then you have to throw it away. (R3:2)”.*

Looking ahead, the participants expressed a desire for additional measures to further reduce the disposal of pharmaceuticals by households. One suggestion was the implementation of systems for the *reuse or recycling* of unopened and unused pharmaceuticals returned to pharmacies.

*“If there were a system to recycle returned medicine instead of just incinerating it, that would be amazing. Then we wouldn't just be sending off everything paid for with tax money to be burned.” (R3:1).*

Regarding disposal from pharmacies, the participants described their efforts to *optimise stock management* to reduce the disposal of pharmaceuticals. They mentioned strategies, such as monitoring inventory levels, to minimise waste. However, many expressed that this was difficult, explaining that pharmacies must maintain large inventories to meet customer demands. This often involves stocking multiple brands of the same pharmaceutical, which increases the risk of pharmaceuticals expiring before they are dispensed. Some participants also mentioned the Swedish system, which mandates pharmacies to dispense a preferred brand, as a significant environmental challenge. They believed this system limits their flexibility and leads to unnecessary waste, as alternative brands with shorter shelf lives may remain unused. A suggestion to reduce the disposal of pharmaceuticals was to introduce greater flexibility in brand selection, allowing pharmacies to dispense pharmaceuticals closer to their expiration dates rather than being restricted to the preferred brand.

*”This system with [the preferred brand] is a bit rigid. Somehow, you should be able to take environmental aspects into it all, too. If I have a lot of a commonly used medicine in stock that is starting to approach its expiration date, then I should be allowed to sell it instead of throwing it away.” (Pm1).*

The participants highlighted that there is no financial incentive for pharmacies to maintain smaller inventories, as they are reimbursed for prescription pharmaceuticals returned due to expiration. Consequently, this reduces the motivation to minimise stock levels and contributes to continued pharmaceutical waste.

*“Since pharmacies, in most cases, get reimbursed for products that have expired on time, there is no financial incentive to look into that issue. Because it doesn't affect the economy.” (Pm 1).*

### 3.2 Limited personal agency, and a need for support

Despite a certain awareness of pharmaceuticals environmental impact, participants struggled to identify concrete measures that could be implemented at the pharmacy level. Instead, they placed much of the responsibility on other stakeholders, arguing that many decisions that would affect the environmental impact of pharmaceuticals are beyond their control. They argued that key environmental decisions must be made at higher levels, such as by *regulatory authorities*. Specifically, they highlighted that meaningful change, such as incorporating environmental requirements into reimbursement systems or considering environmental aspects in the pharmaceutical approval process, must be regulated by decision-making bodies.

*“We don't have much influence when it comes to these things. People will always need medicines. By law, we are required to stock all medicines. If something is prescribed, we have to be able to provide it, even if it's something that ‘kills all the fish,’ or whatever you want to call it. So, it's not like we have a choice in the matter. If the [authorities] have approved it, then we have to supply it.” (R2:1).*

*“A few things are happening, but I think we could do a lot more. But it's hard for us at the pharmacies alone to do it, so we need support from political decisions. And the pharmacy chains need to push these issues more.“(Pm1).*

Participants also felt that *pharmacy chains* could do more, though opinions on their commitment to environmental issues varied. Managers generally perceived environmental efforts as a higher priority for pharmacy chains than non-managers did. Additionally, participants acknowledged the financial pressures from their employers (i.e., pharmacy chains), noting that any substantial engagement with environmental initiatives would require financial incentives.

*“- The company usually says that we do a lot for the environment, but there is a lot more to do (R3:3).*

*- One would really like to know… (R3:2).*

*- Yes, exactly. It sounds like we do a lot for the environment, but when you are standing in the pharmacy and throw away a lot of medicine, it doesn't feel that way. In the end, it's all about money anyway. (R3:3)”.*

*“And it has to be weighed against a cost. If we were to bring in products that are more environmentally friendly and transported in a sustainable way, I think there would be pressure from higher up saying that this is too expensive, that it doesn't add up financially. I find it very hard to believe that this issue is a high priority at higher management levels. My impression is that environmental concerns are not given very high priority when compared to costs.” (R1:6).*

*“We are working very actively on this [environment], and it is a current issue that is always brought up, both among employees and in management meetings.” (Pm4).*

The participants also placed some responsibility on the pharmaceutical industry, emphasising that it is their duty to ensure environmentally safe manufacturing practices.

*“We cannot choose to exclude a medicine just because it is not produced in a sustainable way if it is the medicine the patient needs. Instead, the pharmaceutical companies must put pressure on the manufacturing to ensure that production is done in a better way.” (Pm1).*

Participants also acknowledged that physicians play a crucial role in prescribing practices and have a responsibility to consider the environmental impact of their choices, how much, and what they prescribe.

*“It feels more like it's the responsibility of healthcare, what they prescribe. For example, not prescribing so much diclofenac.” (Pm4).*

Another challenge mentioned was the lack of customer engagement with the environmental aspects of pharmaceuticals. Since customers rarely inquire about these issues, participants found it difficult to initiate discussions on sustainable pharmaceutical use. However, when staff did bring it up, customers were said to respond positively. Some participants suggested that public awareness campaigns could help increase knowledge and encourage more environmentally conscious behaviours among customers.

*“But I don't experience that many people ask about things like that. It's more when you talk about things like ‘Well, is that so?’ Like diclofenac and things like that, they don't have any idea about it at all.” (R1:6).*

*“It could be part of a pharmacy chain's marketing with everything else, like at the same time informing people to think about this, like returning your old medicines.” (R4:2).*

## 4 Discussion

This study showed that community pharmacy staff recognise the environmental impact of pharmaceuticals as an important issue, particularly by expressing concern about the large quantities of pharmaceuticals that are disposed of, and that they wish to do more to address this concern. However, the participants in this study struggled to suggest concrete measures that could be implemented at the pharmacy level. Instead, they placed responsibility on other stakeholders, such as physicians, pharmacy chains, and regulatory authorities. While they believed that more efforts are needed to address the environmental issue, they also perceived it as a difficult challenge due to commercial interests and regulatory barriers.

Participants expressed a clear frustration over the large quantities of pharmaceuticals being disposed of, a concern echoed in previous research.^12^^,^17., 18., 19. In Sweden alone, approximately 1400 t of pharmaceuticals are disposed of from pharmacies each year.^30^ Estimates of the proportion of household pharmaceuticals becoming waste vary widely, from 3 % to as high as 50 % globally. Sweden, Portugal, and the Netherlands report some of the highest rates of returned unused pharmaceuticals, whereas in many countries this proportion is considerably lower.^34^ Research from the UK suggests that up to 50 % of pharmaceutical waste is preventable,^35^ highlighting the need for strategies to minimise unnecessary disposal.

Guidelines, educational initiatives, and improved communication between pharmacists and customers have been shown to significantly reduce pharmaceutical waste.^36^ Controlled clinical trials in the UK and Sweden have shown that pharmaceutical waste can be reduced by up to 30 % when patients starting a new treatment course have the opportunity to discuss their pharmaceutical concerns beyond standard prescribing instructions.^37^^,^^38^ This was also highlighted by participants in this study, who mentioned the importance of customer counselling and the need for increased public awareness through, for example, information campaigns. Proposed strategies to minimise pharmaceutical waste also included allowing for the reuse of unopened packages of pharmaceuticals and dispensing smaller quantities. However, these changes would require legal and regulatory adjustments to be implemented. Currently, strict EU regulations prohibit the reuse or redistribution of returned pharmaceuticals to ensure patient safety and pharmaceutical efficacy. While such regulations are important, they may also contribute to unnecessary waste. In the US, several states have introduced medication recycling programs that facilitate the donation of unused, unopened pharmaceuticals to individuals in need.^39^ Similarly, a recent study from the Netherlands^40^ explored the impact of redistributing oral anticancer pharmaceuticals that were unused by patients. The results showed a 68 % reduction in pharmaceutical waste.

This study also highlights how structural and financial aspects of the Swedish pharmaceutical system can unintentionally contribute to pharmaceutical waste. Notably, Sweden's reimbursement system which grants patients free pharmaceuticals after having paid a certain amount out-of-pocket, may incentivise stockpiling and overdispensing, increasing the risk of waste. Similarly, the system where a specific brand (when generics are available) must be dispensed, is another example, as it restricts pharmacists' flexibility, potentially leaving alternative products with shorter shelf lives unused. Another unintended consequence of this system is when pharmaceuticals regularly change in name, shape, and colour, patients may struggle to recognise their pharmaceuticals, which can lead to non-adherence and unused pharmaceuticals.^41^

Participants felt that pharmacy chains could do more to advocate environmental work in practice, and several questioned the companies' stated ambitions. This perception is consistent with findings from a recent mixed-methods study on Swedish community pharmacies,^23^ which showed that although pharmacy chains have implemented a range of sustainability measures, pharmacy staff often lacked awareness of these. While company-level efforts such as fossil-free deliveries and transparency labelling of over-the-counter pharmaceuticals indicate a commitment to sustainability, the study found that staff had limited knowledge of these initiatives. Together, these results highlight a disconnection between company ambition and the day-to-day practice of community pharmacies.

Another category was that the participants' experiences limited personal agency in addressing pharmaceutical pollution. They attributed their limited agency to structural barriers, reflecting a distancing from personal responsibility. These findings align with a recent study by Zhao et al.^22^ on Canadian community pharmacies, which highlighted that pharmacists often feel powerless regarding influencing environmental practices, unless mandated by external authorities. A voluntary approach to environmentally conscious pharmacy practices, relying solely on individual commitment and goodwill, is unlikely to drive substantial change. Zhao et al. concluded that efficiency and cost-effectiveness consistently took precedence over environmental considerations at the pharmacy level. The competitive nature of the pharmacy sector meant that large financial investments in sustainability initiatives were unlikely unless mandated by regulators.^22^ The findings in this study support this perspective, as the Swedish pharmacy market is also characterised by strong financial competition and many participants (non-managers) expressed feeling pressured by their employers to dispense a high volume of prescriptions, which they perceived as conflicting with considerations of societal costs and environmental impact.

This perceived lack of agency may not only be the case regarding environmental engagement but also relate to broader issues concerning professional identity and empowerment among pharmacists. Previous research on role development has shown that pharmacists often feel they lack the power to effect meaningful change,^42^ indicating that agency is closely tied to how they perceive their professional role, at least in countries where the pharmaceutical market is dominated by chains. Strengthening pharmacists' sense of empowerment or agency is therefore crucial, not only to foster engagement with environmental issues but also to support their professional development more broadly. One way to enhance agency could be through education. In this study, many participants expressed a need for clear and concrete guidance on how they could contribute to reducing pharmaceutical pollution, including access to tools such as a list or database of environmentally harmful pharmaceuticals. The fact that such resources already exist to some extent43., 44., 45. highlights a knowledge gap that could be addressed through education, ensuring that pharmacists have adequate knowledge and tools to act effectively. A recent study on hospital pharmacists in Spain found that, although there is a general awareness of and positive attitude toward the importance of environmental considerations, this awareness is not consistently translated into practice. The study underscored the need for targeted education to bridge the gap between awareness and action.^20^

Overall, there were no major differences in opinions between managers and non-managerial staff. However, managers were generally more positive toward the pharmacy chain's environmental initiatives than non-managerial staff, and tended to adopt a broader strategic and economic perspective. They more often discussed structural challenges in relation to policy. This contrasted with the focus group participants, who largely emphasised day-to-day practices and customer interactions.

This study is subject to limitations. Self-selection may have influenced which individuals chose to participate, potentially skewing the sample toward those with higher interest in environmental issues. A key consideration is the sampling strategy, as pharmacy staff from different settings may have varying perspectives on the role of pharmacies in reducing pharmaceutical pollution. Furthermore, the sample was predominantly female and pharmacy-educated, which may limit the perspectives represented. At the same time, a huge majority of pharmacy staff in Sweden are pharmacy-educated and women. No pharmacy staff from online or independent pharmacies were included. However, online pharmacies have less direct contact with customers, and the independent pharmacies are very few. Including different types of pharmacy staff provided deep insights into the practical, organisational and strategic aspects of pharmacies' environmental work.

A common challenge was scheduling the focus groups, as finding a suitable time for all participants proved difficult. Some participants had to cancel at the last minute, which resulted in small group sizes and may have impacted group dynamics and the range of perspectives represented in the discussions.

Moreover, the qualitative analysis could have been influenced by the researchers' preunderstandings and interpretations. To enhance the reliability of the findings, the content analysis was conducted by two researchers with different backgrounds and experience in qualitative methods, and the assistant moderator, who had experienced the focus group/interviews, audited the results.

## 5 Conclusion

This study highlights important perspectives on the role of community pharmacies in reducing pharmaceutical pollution and the expressed willingness of staff to contribute to improvements. Pharmacy staff struggled to identify concrete actions at the pharmacy level and often shifted responsibilities to physicians, pharmacy chains, and regulatory authorities. Pharmaceutical waste emerged as a key concern, exacerbated by structural aspects and a lack of regulatory flexibility. The pharmacy staff felt limited in their ability to address pharmaceutical pollution, as they viewed meaningful change as dependent on actions from higher levels, such as pharmacy chains or regulators. To enable more proactive engagement, national, industry-wide requirements must be complemented by targeted education and training that strengthen pharmacy staff's sense of agency and equip them with the knowledge and confidence to act.

## CRediT authorship contribution statement

**Johanna Villén:** Writing – original draft, Project administration, Methodology, Investigation, Formal analysis, Data curation, Conceptualization. **Nicole Ljungdahl:** Writing – review & editing, Validation, Methodology, Data curation. **Björn Wettermark:** Writing – review & editing, Methodology, Conceptualization. **Helle Håkonsen:** Writing – review & editing, Methodology, Conceptualization. **Marmar Nekoro:** Writing – review & editing, Methodology, Conceptualization. **Sofia Kälvemark Sporrong:** Writing – review & editing, Supervision, Methodology, Formal analysis, Conceptualization.

## Declaration of competing interest

The authors declare that they have no known competing financial interests or personal relationships that could have appeared to influence the work reported in this paper.

## Appendix A Supplementary data

Supplementary material: Interview guide and overview of conducted interviews. Image 1

## References

[1] Boxall A.B. (2004). The environmental side effects of medication. EMBO Rep.

[2] Daughton C.G., Ruhoy I.S. (2009). Environmental footprint of pharmaceuticals: the significance of factors beyond direct excretion to swewers. Environ Toxicol Chem.

[3] Wellington E.M., Boxall A.B., Cross P. (2013). The role of the natural environment in the emergence of antibiotic resistance in gram-negative bacteria. Lancet Infect Dis.

[4] Oaks J.L., Gilbert M., Virani M.Z. (2004). Diclofenac residues as the cause of vulture population decline in Pakistan. Nature.

[5] Jackson L.M., Felgenhauer B.E., Klerks P.L. (2019). Feminization, altered gonadal development, and liver damage in least killifish (Heterandria formosa) exposed to sublethal concentrations of 17α-ethinylestradiol. Ecotoxicol Environ Saf.

[6] Zhou X., Li Y., Li H., Yang Z., Zuo C. (2019). Responses in the crucian carp (Carassius auratus) exposed to environmentally relevant concentration of 17α-Ethinylestradiol based on metabolomics. Ecotoxicol Environ Saf.

[7] Kvarnryd M., Grabic R., Brandt I., Berg C. (2011). Early life progestin exposure causes arrested oocyte development, oviductal agenesis and sterility in adult Xenopus tropicalis frogs. Aquat Toxicol.

[8] Daughton C.G. (2003). Cradle-to-cradle stewardship of drugs for minimizing their environmental disposition while promoting human health. I. Rationale for and avenues toward a green pharmacy. Environ Health Perspect.

[9] Daughton C.G., Ruhoy I.S. (2011). Green pharmacy and pharmEcovigilance: prescribing and the planet. Expert Rev Clin Pharmacol.

[10] Roy C. (2021). The pharmacist's role in climate change: a call to action. Can Pharm J CPJ.

[11] Lunghi C., Valetto M.R., Caracciolo A.B. (2024). Call to action: pharmaceutical residues in the environment: threats to ecosystems and human health. Drug Saf.

[12] Afanasjeva J., Gruenberg K. (2019). Pharmacists as environmental stewards: strategies for minimizing and managing drug waste. Sustain Chem Pharm.

[13] (2016). FIP Statement of Policy - Environmentally Sustainable Pharmacy Practice.

[14] Royal Dutch Pharmacists Association (KNMP) (2025). Green Pharmacy Toolkit Tips and Tricks Royal Dutch Pharmacists Association. https://sustainability.fip.org/wp-content/uploads/2024/09/Green-Pharmacy-Groene-Apotheek-Engelstalig-def.pdf.

[15] Michael I., Ogbonna B., Sunday N., Anetoh M., Matthew O. (2019). Assessment of disposal practices of expired and unused medications among community pharmacies in Anambra State southeast Nigeria: a mixed study design. J Pharm Policy Pract..

[16] Asmamaw G., Agedew T., Tesfaye B. (2023). Prevalence of leftover medicines, disposal practices, and associated factors in Arba Minch Town, Southern Ethiopia. SAGE Open Med.

[17] Bekker C.L., Gardarsdottir H., Egberts A.C.G., Bouvy M.L., BJF Bemt (2018). Pharmacists' activities to reduce medication waste: an international survey. Pharmacy.

[18] Smale E.M., Van Der Werff I.B., Johannes B., Van Den Bemt F., Linde Bekker C. (2023). How to engage healthcare providers in preventing medication waste through individualized prescribing and dispensing: a qualitative study. Res Soc Adm Pharm.

[19] Smale E.M., Egberts T.C.G., Heerdink E.R., van den Bemt B.J.F., Bekker C.L. (2021). Waste-minimising measures to achieve sustainable supply and use of medication. Sustain Chem Pharm.

[20] Domingo-Echaburu S., Abajo Z., Elizondo-Alzola U. (2025). Knowledge, attitude, and practice about the environmental impact of pharmaceuticals among hospital pharmacists in Spain: a cross-sectional survey. Int J Pharm Pract.

[21] Singleton J.A., Lau E.T.L., Nissen L.M. (2018). Waiter, there is a drug in my soup – using Leximancer® to explore antecedents to pro-environmental behaviours in the hospital pharmacy workplace. Int J Pharm Pract.

[22] Zhao A., Gregory P.A.M., Austin Z. (2024). Climate-conscious pharmacy practice: an exploratory study of community pharmacists in Ontario. Can Pharm J.

[23] Salehi E., Ljungberg Persson C., Håkonsen H. (2025). Green pharmacy practice – a multi method study of environmental sustainability measures implemented in Swedish pharmacies. J Pharm Policy Pract.

[24] Krueger R., Casey M., Focus Groups (2009).

[25] Tong A., Sainsbury P., Craig J. (2007). Consolidated criteria for reporting qualitative research (COREQ): a 32-item checklist for interviews and focus groups. Int J Qual Heal Care.

[26] Westerlund T., Söderlund L.Å. (2023). Role of community pharmacy and pharmacists in self care in Sweden. Explor Res Clin Soc Pharm.

[27] Håkonsen H., Sundell K.A., Babar Z.U.D. (2015). Pharmaceutical Prices in the 21st Century.

[28] Janlöv N., Blume S., Glenngård A.H., Hanspers K., Anell A., Merkur S. (2023). Sweden: Health System Review. https://iris.who.int/bitstream/handle/10665/372708/9789289059473-eng.pdf?sequence=8.

[29] Sveriges riksdag SFS 2009:366. Lag Om Handel Med Läkemedel [in Swedish]. https://www.riksdagen.se/sv/dokument-och-lagar/dokument/svensk-forfattningssamling/lag-2009366-om-handel-med-lakemedel_sfs-2009-366.

[30] Swedish Pharmacy Association (2024). Sector Report. https://sverigesapoteksforening.se/wp-content/uploads/2024/05/Eng-version-final-3.pdf.

[31] (2021). Introducing Whisper. https://openai.com/index/whisper/.

[32] Malterud K., Siersma V.D., Guassora A.D. (2016). Sample size in qualitative interview studies: guided by information power. Qual Health Res.

[33] Traulsen J.M., Almarsdóttir A.B., Björnsdóttir I. (2004). Interviewing the moderator: an ancillary method to focus groups. Qual Health Res.

[34] OECD (2022).

[35] Trueman P., Lowson K., Blighe A. (2010).

[36] OECD (2017).

[37] Clifford S., Barber N., Elliott R., Hartley E., Horne R. (2006). Patient-centred advice is effective in improving adherence to medicines. Pharm World Sci.

[38] Schedlbauer A., Schroeder K., Fahey T. (2007). How can adherence to lipid-lowering medication be improved? A systematic review of randomized controlled trials. Fam Pract.

[39] National Conference of State Legislatures (2024). State Prescription Drug Repository Programs. https://www.ncsl.org/health/state-prescription-drug-repository-programs.

[40] Smale E.M., BJF Bemt, Heerdink E.R. (2024). Cost savings and waste reduction through Redispensing unused Oral anticancer drugs: the ROAD study. JAMA Oncol.

[41] Håkonsen H., Eilertsen M., Borge H., Toverud E.L. (2009). Generic substitution: additional challenge for adherence in hypertensive patients?. Curr Med Res Opin.

[42] Svensberg K., Kälvemark Sporrong S., Håkonsen H., Toverud E.L. (2015). ‘Because of the circumstances, we cannot develop our role’: Norwegian community pharmacists' perceived responsibility in role development. Int J Pharm Pract..

[43] Ramström H., Martini S., Borgendahl J., Ågerstrand M., Lärfars G., Ovesjö M.L. (2020). Pharmaceuticals and environment: a web-based decision support for considering environmental aspects of medicines in use. Eur J Clin Pharmacol.

[44] Fass (2025). Information till vårdpersonal [Information to health care professionals]. https://www.fass.se/LIF/startpage?userType=0.

[45] Linder E., Villén J., Nekoro M., Wettermark B., Kälvemark Sporrong S. (2023). Stakeholders' perspectives and use of web-based knowledge support for environmental information on pharmaceuticals. Explor Res Clin Soc Pharm..

