# Supplementary material for: “We throw away an incredible amount of unused medicines“: Community pharmacy staff perspectives on environmental responsibility
Source: Explor Res Clin Soc Pharm. 2025 Sep 19;20:100662. doi: 10.1016/j.rcsop.2025.100662 (PMC12510222; doi:10.1016/j.rcsop.2025.100662)
Supplement: Supplementary file 1 — Supplementary material: Interview guide and overview of conducted interviews. [file mmc1.docx]

Supplementary material

**Supplementary table 1. Semi-structured interview guide**

| 1. **Can you tell me what comes to mind when you hear the phrase “pharmaceuticals and the environment”?** |
| --- |
| - Do you think it’s important? - Is it something you’ve thought about before? |
| 1. **Can you tell me about how you currently work with pharmaceuticals and the environment in your pharmacy?** |
| - How is this work beneficial for the environment? |
| 1. **What experiences have you had with customers asking questions about the environmental or climate impact of pharmaceuticals?** |
| - What kinds of questions do they ask, and how do you respond? |
| 1. **How could pharmacies do more to reduce the environmental problems associated with pharmaceuticals?** |
| - Imagine you had complete decision-making power—what would you do? - For example, would you make changes to:   - The product range?   - Placement of products in the store?   - Customer advice?   - Disposal of unused pharmaceuticals? - Are there differences in the actions you could or should take regarding prescription pharmaceuticals versus over-the-counter pharmaceuticals? |
| 1. **What do you think pharmacies’ responsibilities are compared to other actors or professions?** |
| - For example, compared to doctors/prescribers? - The pharmaceutical industry? - Regulatory authorities? - How could pharmacies collaborate with others, such as:   - Other professions?   - Other actors in society? |
| 1. **Do you see any challenges or barriers to pharmacies working more actively with pharmaceuticals and the environment?** |
| - Knowledge:   - What information have you received about the environmental impact of pharmaceuticals, and from what sources?   - What knowledge is needed or missing?   - Do you think there are ways to increase pharmacy staff awareness and knowledge about the environmental impact of pharmaceuticals? - Resources:   - How do you think your pharmacy chain prioritises sustainability compared to other concerns (e.g., profitability)? |
| 1. **How do you view the balance between considering the environment and always prioritising the customer’s best interest?** |
| - Could there be situations where these two perspectives conflict? - If so, how do you approach or think about these conflicts? |
| 1. **Is there anything you would like to discuss that we haven’t discussed today?** |

**Supplementary table 2. Overview of conducted interviews (listed in chronological order)**

| **Type of interview** | **How?** | **Participants** | **Duration of the interview in minutes** |
| --- | --- | --- | --- |
| Focus group | In person | Six women | 102 |
| Focus group | In person | Three men | 90 |
| Individual interview | Digital | One woman | 25 |
| Individual interview | Digital | One woman | 34 |
| Focus group | Digital | Three women | 76 |
| Focus group | Digital | Two women | 50 |
| Individual interview | Digital | One woman | 37 |
| Individual interview | Digital | One woman | 29 |
| Individual interview | In person | One man | 34 |
